# Research Progress on the Cardiovascular Protective Effect of Glucagon-Like Peptide-1 Receptor Agonists

**DOI:** 10.1155/2022/4554996

**Published:** 2022-04-08

**Authors:** Rui Song, Hang Qian, Yunlian Wang, Qingmei Li, Dongfeng Li, Jishun Chen, Jingning Yang, Jixin Zhong, Handong Yang, Xinwen Min, Hao Xu, Yong Yang, Jun Chen

**Affiliations:** ^1^Sinopharm Dongfeng General Hospital, Hubei University of Medicine, Shiyan, Hubei 442000, China; ^2^Department of Immunology, School of Basic Medicine, Hubei University of Medicine, Shiyan, Hubei 442000, China; ^3^Department of Rheumatology and Immunology, Tongji Hospital, Huazhong University of Science and Technology, Wuhan, Hubei 430030, China; ^4^Hubei Key Laboratory of Wudang Local Chinese Medicine Research (Hubei University of Medicine), China; ^5^Institute of Virology, Hubei University of Medicine, Shiyan, Hubei 442000, China

## Abstract

The risk of cardiovascular diseases is closely related to diabetes. Macrovascular disease is the main cause of death and disability in patients with type 2 diabetes. In recent years, the glucagon-like peptide-1 receptor agonist (GLP-1RA), a new type of hypoglycemic drug, has been shown to regulate blood sugar levels, improve myocardial ischemia, regulate lipid metabolism, improve endothelial function, and exert a protective role in the cardiovascular system. This study reviewed the protective effects of GLP-1RA on the cardiovascular system.

## 1. Introduction

The prevalence of type 2 diabetes (T2DM) continues to increase every year. The International Diabetes Federation data predicts that the number of patients worldwide will reach 629 million by 2045 [1]. Diabetes-associated complications are mainly divided into microvascular disease and macrovascular disease. Macrovascular disease mainly manifests as atherosclerotic disease, which is the main cause of death and disability in patients with T2DM. The occurrence and development of atherosclerosis (AS) are caused by multiple factors, including smoking, hypertension, hyperlipidemia, hyperuric acid, hyperglycemia, and other factors [2, 3]. Glucagon-like peptide-1 receptor agonist (GLP-1RA) is a new type of drug for treating diabetes. Studies have shown that GLP-1RA can exert hypoglycemic and cardiovascular protective effects. This study reviewed the protective effects of GLP-1RA on the cardiovascular system and provided a theoretical basis for the prevention and treatment of cardiovascular diseases.

### 1.1. Incretin System

Incretin involves the direct stimulation of intestinal epithelial cells by nutrients, leading to the secretion of glucose-dependent insulinotropic polypeptide and glucagon-like peptide-1 (GLP-1), which promotes an insulin secretion. GLP-1 is a glucose concentration-dependent enteropeptidtide hormone encoded by the glucagon gene secreted by L cells of the jejunum, ileum, and colon, which cleaves into two different existing forms of GLP-1 after cleavage by proprotein converting 1(PC1). The first form is the amidated form of GLP-1 consisting of 30 amino acids, namely, GLP-1 (7-36) amide; the second form is the 31-peptide form extending into the glycine, namely, GLP-1 (7-37). Most of the effective GLP-1 is dissolved in the form of GLP-1 (7-36) amide: GLP-1:78-107 and 78-108 [4].

GLP-1 specifically binds to the glucagon-like peptide-1 receptor (GLP-1R) in the body, directly stimulating the secretion of insulin by pancreatic *β*-cells, promoting proliferation and differentiation, and inhibiting cell apoptosis, thereby exerting a hypoglycemic effect. In addition, GLP-1 also participates in blood sugar regulation by reducing glucagon levels, delaying gastric emptying, increasing satiety, and reducing appetite [5]. GLP-1R is a G protein–coupled receptor widely distributed in the pancreas, lung, heart, kidney, vascular smooth muscle, fat cells, gastrointestinal tract, central nervous system, and other tissues [6]. GLP-1 recognizes and binds to GLP-1R, activating GLP-1R, upregulating the cyclic adenosine monophosphate (AMP) level and intracellular Ca^2+^ concentration in the body and subsequently releasing glucose-dependent insulin, thereby protecting the function of islets and regulating blood sugar levels. The natural GLP-1 in the human body is easily degraded by dipeptidyl peptidase-IV (DPP-IV) and can lose its activity quickly. Glucagon-like peptide-1 receptor agonist (GLP-1RA) works by simulating natural GLP-1 to activate GLP-1A. It is not easily degraded by DPP-IV with prolonged half-life and increased concentration of active GLP-1 in the body. The products of GLP-1RA include liraglutide, exenatide, albiglutide, losenatide, dulaglutide, and semaglutide.

GLP-1R is widely expressed in the cardiovascular system, muscle, fat, liver, and other tissues. It is involved in intracellular metabolism and signal transduction. These metabolites have biological activities. They can reduce intravascular oxidative stress, protect the cardiovascular system, increase the vitality of myocardial cells, improve heart function, promote vasodilation, protect pancreatic *β*-cells, and inhibit hepatocyte gluconeogenesis and oxidative stress, thereby directly or indirectly playing a protective role in the cardiovascular system [7].

### 1.2. Direct Protective Effect of GLP-1RA on the Cardiovascular System

#### 1.2.1. Impact on AS

AS is the pathological basis of diseases such as coronary heart disease (CHD), peripheral arterial disease (PAD), and cardiovascular desease (CVD). The inflammatory response induced by the adhesion of monocytes and vascular wall damage plays an important role in the early stages of AS. Studies have found that, besides lowering blood sugar levels, GLP-1RA also has an antiatherosclerotic effect. GLP-1RA reduces the infiltration of inflammatory cells, inhibits the release of inflammatory factors, and improves oxidative stress, thereby reducing the damage to the cell endothelium, improving endothelial cell function, and inhibiting the occurrence of AS [8]. In the atherosclerotic lesions, the use of GLP-1RA inhibited further vascular intimal macrophage infiltration, calcium deposition, extra cellular matrix (ECM) remodeling, and vascular smooth muscle cell (VSMC) proliferation, acting to limit further thickening and plaque rupture [9].

During oxidative stress, platelet overactivity has an important influence on the risk of atherosclerotic thrombotic events. The effect of GLP-1RA on mitochondria not only changes fatty acid oxidation and energy consumption but also has antiapoptotic and antioxidant effects [10]. Exenatide inhibits the expression of inflammatory markers such as high-sensitivity C-reactive protein and monocyte chemoattractant protein-1 in patients with AS, thereby reducing the damage caused by oxidative stress and inflammatory response to vascular endothelial cells [11]. In addition, exenatide increases the release of cyclic adenylate and further inhibits platelet aggregation induced by thrombin, adenosine diphosphate, or collagen [12]. In the AS model induced in Apoe^−/−^ mice, the administration of GLP-1RA effectively inhibited the progression of early-onset, low-load atherosclerotic disease [13]. Jojima et al. [14] observed that the liraglutide induced a cell cycle arrest by activating the AMP-activated protein kinase (AMPK) signal and inhibited the proliferation of vascular smooth muscle cells induced by angiotensin II, thereby delaying AS progression, which had nothing to do with the hypoglycemic effect. Moreover, it also reduced the expression of vascular cell adhesion molecule (VCAM) and intercellular adhesion molecule-1 in human vascular endothelial cells and inhibited the development of AS. The mechanism might be related to the inhibition of pancreatic *β*-cell apoptosis, increase in insulin synthesis, and reduction in glycogen decomposition.

#### 1.2.2. Impact on Heart Failure

The role of GLP-1RA in heart failure is still controversial. Clinical studies have shown that the administration of GLP-RA for 5 weeks could improve the left ventricular ejection fraction and 6-min walking distance and increase the maximum oxygen uptake (VO_2_ max) in patients with heart failure [15]. GLP-1RA improves the insulin receptor in cardiomyocytes, increases glucose uptake and utilization, promotes the recovery of coronary blood flow, and reduces the level of atrial natriuretic peptide and peripheral vascular resistance, thereby exerting a cardiovascular protective effect in patients with heart failure [16].

Matsubara et al. [17] established an animal myocardial ischemia–reperfusion model and showed that the infusion of GLP-1 and human transferrin could significantly prolong the half-life of the drug and improve the wall motion score index and the left ventricular ejection fraction, confirming that GLP-1 intervention could significantly reduce the infarct size, improve the wall motion index and left ventricular ejection fraction after reperfusion, and ameliorate myocardial reperfusion injury. GLP-1RA can slightly increase the resting heart rate of patients with heart failure and reduce the heart rate variability, thereby improving the outcome of cardiovascular diseases [18]. The administration of albiglutide for 12 weeks can improve the oxygen consumption of cardiomyocytes in patients with heart failure [19]. Chen et al. [20] observed that the left ventricular ejection fraction was slightly improved and the markers of inflammation and endothelial function improved with the short-term use of liraglutide in patients with non-ST-segment elevation myocardial infarction (NSTEMI). The use of exenatide improved the diastolic function of patients with T2DM and reduced the degree of AS [21]. A meta-analysis including 29,034 patients with diabetes treated with GLP-1RA showed that GLP-1RA reduced cardiovascular mortality and heart failure hospitalization rate [22]. A retrospective study by Velez et al. [23] showed that GLP-1RA reduced the incidence of heart failure by 49% (95% confidence interval 0.34–0.77, *P* = 0.02). In addition, the exenatide caused no significant difference in the risk of heart failure hospitalization compared with placebo [24]. However, the use of liraglutide significantly reduced cardiovascular mortality, nonfatality myocardial infarction, and nonfatal stroke compared with placebo, but the risk of hospitalization for heart failure was reduced by 13% [25]. As an adjuvant treatment for patients with heart failure, GLP-1RA still requires further basic experiments and clinical studies for validation.

#### 1.2.3. Impact on Myocardial Infarction

The protective effect of GLP-1 receptor agonists on ischemic myocardium works through the AMPK/phosphoinositide 3-kinase (PI3K)–protein kinase B (Akt) pathway [26]. GLP-1RA activates cAMP through PI3K, thereby inhibiting the expression of apoptotic factors and ultimately inhibiting cardiomyocyte apoptosis and improving cardiac function [27].

Sassoon et al. [28] established an animal model of myocardial infarction (MI) and showed that the activation of GLP-1 effectively reduced the expression of the *β*1-adrenergic receptor in myocardial tissues, increased diastolic function and cardiac output, and reduced myocardial oxygen consumption. After MI occurs, GLP-1 induces human heart fibroblasts to produce new elastic fibers, restricts the expansion of the heart, and plays a beneficial role in the recovery of heart function [29]. In patients with NSTEMI, direct percutaneous coronary intervention can be performed, and exenatide adjuvant therapy during reperfusion can increase myocardial ischemia–reperfusion, thereby showing cardioprotection. Meanwhile, Lilaru peptides also have similar effects [30]. In a double-blind trial, the follow-up of patients with T2DM having a high risk of CVD showed that the incidence of death from cardiovascular diseases and stroke in patients taking liraglutide was significantly lower than that in the placebo group [25]. Lonborg et al. [31] found that the postoperative cardiac magnetic resonance examination in patients with myocardial infarction, percutaneous coronary intervention, and continuous infusion of exenatide for 6 h showed that the MI area was reduced compared with earlier. The evaluation after 90 days showed that the intervention effectively reduced the degree of myocardial necrosis and improved postoperative heart function. A meta-analysis showed that GLP-1RA reduced the relative risk of MI by 9%, stroke by 14% and cardiovascular death by 12% [32]. A recent study found that the incidence of major cardiovascular events in patients treated with semaglutide was significantly lower than that in patients treated with placebo [33].

It has been confirmed that liraglutide, semaglutide, and dulaglutide all have cardiovascular protective effects. In 2020, the American Diabetes Association released the “Cardiovascular Disease and Cardiovascular Risk Management,” which pointed out that GLP-1RA with cardiovascular benefits was recommended for patients with coronary heart disease or multiple risk factors for coronary heart disease [34].

### 1.3. Indirect Protective Effect of GLP-1RA on the Cardiovascular System

Cardiovascular diseases are associated with vascular inflammation, endothelial dysfunction, and oxidative stress [35]. GLP-1 receptor agonists inhibit the proliferation of vascular smooth muscle cells and vascular endothelial cells, reduce oxidative stress, promote the increase in the production of nitric oxide (NO), and increase microvascular blood flow, which have beneficial cardiovascular outcomes [36].

#### 1.3.1. Blood Pressure

The mechanism of GLP-1 receptor agonists in lowering blood pressure is not yet fully understood, but the possible mechanisms include changes in the nervous system or changes in the vasopressin regulatory system [9]. According to UKPDS research, liraglutide and exenatide can lower blood pressure by 1-5 mm Hg. The antihypertensive effect is probably exerted via directly activating GLP-1R in the arterial and renal systems, improving vascular endothelial function, and inhibiting the renin–angiotensin–aldosterone system (RAAS), thereby playing a role in vasodilation and natriuresis. Other mechanisms may be the activation of NO by guanosine monophosphate, exerting a vasodilator effect and lowering blood pressure [37].

Tonneijck et al. [38] pointed out that the continuous use of GLP-1 receptor agonists for 8 weeks significantly increased renal sodium excretion, which might be one of the important factors for lowering blood pressure. Animal studies have found that GLP-1 receptors are widely expressed in the atria and GLP-1 receptor agonists can reduce blood pressure by increasing the secretion of atrial natriuretic factors [39]. Many studies found that GLP-1 RA drugs lowered blood pressure. The meta-analysis conducted by Wang et al. [40] showed that GLP-1 receptor agonists lowered blood pressure. Compared with placebo, exenatide and liraglutide reduced systolic and diastolic blood pressures by 1–5 mm Hg (1 mm Hg = 0.133 kPa) in patients with T2DM. A randomized clinical trial showed that liraglutide reduced systolic blood pressure by 2–7 mm Hg in patients with T2DM [41]. A similar antihypertensive effect was also observed in the clinical trials of exenatide [42, 43]. In the study by Maringwa et al. [44], the administration of GLP-1 receptor agonists in patients with diabetes and obesity could reduce systolic blood pressure by 2.8 mm Hg on average without any significant effect on diastolic blood pressure. A study by Ferdinand et al. [45] showed that compared with placebo, dulaglutide could significantly reduce 24-h systolic blood pressure. The changes in diurnal systolic blood pressure indicated that dulaglutide also reduced day and night systolic blood pressure. However, no differences of day or night diastolic blood pressure were found between the two groups.

#### 1.3.2. Blood Lipids

Hyperlipidemia is the main risk factor for arteriosclerotic cardiovascular disease [46]. Besides increasing insulin secretion, GLP-1 receptor agonists can improve the level of lipid metabolism in patients. They also inhibit liver peroxidase *β* oxidation, reduce the intestinal absorption of lipids in food, reduce triglyceride level after meals, and induce the decrease in LDL-C levels by enhancing liver fatty acid oxidation [47, 48].

In a randomized controlled clinical study, patients with T2DM, who used GLP-1 receptor agonists to reduce the blood sugar level, showed significantly reduced levels of triglyceride (TC), Apo B-48, and Apo C-III [26]. Wismann et al. [49] confirmed that the decreased secretion of chylomicrons in intestinal epithelial cells might be caused by GLP1R, which is mainly distributed in intestinal neurons and lymphocytes and indirectly mediated by nerve signals. Liraglutide and exenatide reduced postprandial blood lipid levels 2 weeks after the initial treatment, which had nothing to do with gastric emptying [50]. An 18-month prospective study found that waist circumference, body mass index, fasting blood glucose, glycosylated hemoglobin, total cholesterol and low-density lipoprotein cholesterol, triglycerides, and carotid artery intima-media thickness all improved after 18 months of liraglutide treatment in overweight and obese patients [51]. A previous study showed that after administering exenatide into a mouse model of hyperlipidemia, the levels of free fatty acids, triglycerides, and leptin significantly reduced [52]. Nauck et al. [53] observed that the protective effect of GLP-1 receptor agonists on the cardiovascular system did not depend on weight loss, reduction of glycosylated hemoglobin level, and severe hypoglycemia.

These data indicated that GLP-1 receptor agonists improved dyslipidemia and protected the cardiovascular system by regulating the body's metabolic disorders by reducing postprandial blood lipids and blood sugar, improving myocardial and vascular inflammation, controlling blood pressure, and reducing weight [9].

#### 1.3.3. Blood Sugar

Diabetes is closely related to the occurrence of cardiovascular diseases. The risk of cardiovascular and cerebrovascular diseases increases by two to four times in patients with diabetes than in nondiabetic patients. A prospective study in the United Kingdom showed that the early control of blood sugar in patients with diabetes significantly reduced the incidence of cardiovascular events and mortality [54]. GLP-1 binds to the GLP-1 receptor on pancreatic *β*-cells and directly stimulates the secretion of insulin from pancreatic *β*-cells in a glucose-dependent manner. It also promotes the proliferation of pancreatic *β*-cells, inhibits apoptosis, increases the number of *β*-cells, and promotes insulin synthesis, thus playing a hypoglycemic effect [5]. GLP-1 receptor agonists can increase glucose-dependent insulin secretion, reduce the level of glucagon, and reduce fasting and 2-h postprandial blood sugar levels.

Besides lowering blood sugar levels, GLP-1RA can improve insulin sensitivity by improving endocrine and metabolic disorders such as hyperlipidemia and obesity, increasing glucose uptake by peripheral tissues, and exerting a hypoglycemic effect. Recent studies have found that GLP-1RA can reduce the endogenous glucose level, but its specific mechanism needs to be further investigated. Seghieri et al. [55] found that the changes in insulin and glucagon levels did not affect the influence of GLP-1 on inhibiting endogenous glucose production. Baggio et al. [56] showed that GLP-1RA acted on pancreatic islet *α* cells and inhibited the release of glucagon, thereby reducing liver glucose production. GLP-1 receptor agonists specifically bind to GLP-1 receptors in nerve afferent nerves to inhibit gastrin and gastric acid secretion after eating, delay gastric emptying, and reduce postprandial blood sugar levels [57, 58].

In addition, GLP-1 receptor agonists also participate in blood glucose regulation by enhancing glucose utilization in peripheral tissues, increasing satiety, and reducing appetite. They have the characteristics of blood glucose–dependent effects, leading to a low chance of hypoglycemia [59]. Current studies have found that fasting blood glucose, postprandial blood glucose, and glycosylated hemoglobin levels all improved after GLP-1RA treatment in patients with diabetes [60]. Short-acting GLP-1RA preparations mainly affect postprandial blood glucose levels, while long-acting GLP-1RA can reduce fasting and postprandial blood glucose levels.

The hypoglycemic mechanism of GLP-1RA is more complicated, mainly through specific binding with GLP-1 receptors in the central, liver, muscle, adipose, and other tissues to work together to play a hypoglycemic effect.

#### 1.3.4. Vascular Endothelium

Driven by vascular endothelial inflammation, ECM remodels, vascular smooth muscle cell activation, proliferation and migration, macrophage infiltration, and foam cell formation contribute to AS [61].

Animal studies have shown that liraglutide promotes the expression of vascular endothelial nitric oxide synthase, reduces intercellular adhesion molecule-1, and improves mouse vascular endothelium. Exendin-4 attenuates the expression of plasminogen activator inhibitor type 1(PAI-1) and VCAM in human vascular endothelial cells *in vitro*, increases ROS levels, promotes NO production, and reverses homocysteine acid–induced endothelial dysfunction [62]. Lnborg et al. [31] constructed a myocardial ischemia–reperfusion model and observed that exenatide activated adenosine triphosphate–sensitive potassium channels to protect endothelial dysfunction induced by ischemia–reperfusion. Aya Shiraki et al. [63] showed that liraglutide had an antioxidant and antiinflammatory effect on vascular endothelial cells. It inhibited tumor necrosis factor-*α–* (TNF-*α*–) induced oxidative stress damage of endothelial cells by increasing the expression of catalase, superoxide dismutase-2(SOD-2), and glutathione peroxidase (GPX) protein levels. Chang et al. [64] showed that the liraglutide reduced the damage caused by oxidized low-density lipoproteins to vascular endothelium by inhibiting the activation of p53.

GLP-1 RA can activate GLP-1R and AMPK, induce vasodilation, and reduce vascular endothelial dysfunction caused by hyperglycemia or hyperlipidemia probably through improving metabolism and directly acting via vascular mechanisms [65].

#### 1.3.5. Weight Loss

Weight control can significantly reduce the occurrence of cardiovascular events, and GLP-1 RA has the effect of reducing body mass. GLP-1 acts on the hypothalamic paraventricular nucleus, arcuate nucleus, and lateral hypothalamus to suppress appetite, reduce food consumption, inhibit gastric emptying, and increase satiety, thereby increasing gastric dilatation and reducing gastric acid secretion [66, 67]. Cirincione et al. [68] showed that GLP-1 receptor agonists easily passed through the blood–brain barrier and directly acted on the center of satiety and food intake through the vagus nerve-dependent and independent pathways, thereby suppressing appetite. Current studies have found that GLP-1R receptor agonists reduce weight in a variety of ways, delay gastric emptying, reduce appetite and hunger, and increase satiety, thereby reducing body weight [69, 70].

In pharmacological trials, GLP-1RA has been shown to delay gastric emptying in the first hour after a meal. Coveleski et al. [71] found that the use of GLP-1RA increased the connection between the hypothalamus and thalamus and the nucleus tractus solitarii, thereby reducing hunger and food intake and promoting weight loss. Studies in obese mouse models have shown that GLP-1RAs can bind to GLP-1 receptors in specific brain regions related to appetite regulation, leading to the stimulation of proopiomelanocortin (POMC)/cocaine- and amphetamine-regulated transcript (CART) neurons, inhibition of neuropeptide Y, increased satiety, reduced hunger, and subsequent reduction in energy intake, thus playing a role in weight loss [72]. Animal studies have shown that GLP-1RA specifically binds to the GLP-1 receptor in the central system to stimulate the thermogenesis of brown adipose tissue and the browning of white adipose tissue, reduce food intake, and increase energy consumption [73]. VAN CAN et al. [70] observed that the GLP-1RA treatment in obese patients significantly reduced energy intake and appetite, especially for high-fat foods. The continuous use of liraglutide can reduce body weight by 2–3 kg, which is considered to be related to slowing down gastric emptying, promoting satiety, and reducing food intake.

### 1.4. Cardiovascular Risk with GLP-1RA

Due to the short half-life of natural GLP1, existing research is aimed at developing GLP-1RA with longer half-life. GLP-1RAs can be classified into short-acting (exenatide and lixisenatide) and long-acting (liraglutide, albiglutide, dulaglutide, and semaglutide) according to the duration of their action on the receptor. Cardiovascular disease (CVD) is a common and serious comorbidity of type 2 diabetes mellitus (T2DM), and compulsory cardiovascular outcome trials (CVOTs) have become an important evaluating new antidiabetes drug development [74]. Several studies published to date demonstrated statistical evidence of CV benefit of GLP1RA.The LEADER CVOT with liraglutide was the first study to show cardioprotection effect of GLP-1 [25]. Fewer patients occurred a primary outcome in the liraglutide group than in the placebo group (13.0% vs. 14.9%, hazard ratio, 0.87; 95% confidence interval, 0.78-0.97; *P* < 0.001 for noninferiority; *P* = 0.01 for superiority). The EXSCEL (Exenatide Study of Cardiovascular Event Lowering) trial published in 2017 is the largest (*N* = 14,752) CVOT with GLP-1RAs to date. Exenatide ER revealed a statistical noninferiority versus placebo in the primary outcome of major adverse cardiovascular events (HR 0.91; 95% CI, 0.83-1; *P* < 0.001 for noninferiority) and no statistical superiority (*P* = 0.06) [24]. The SUSTAIN 6 study, published in 2016, was a trail to evaluate the cardiovascular and other long-term outcomes with semaglutide (subcutaneous injection once-weekly of 0.5 mg or 1.0 mg) (*N* = 3,297) [33]. A post hoc analysis of the sustain 6 trial demonstrated superiority of once-weekly semaglutide 0.5 mg or 1 mg versus placebo for the primary composite MACE (CV death, nonfatal MI, or nonfatal stroke) endpoint (*P* = 0.02). CVOT of oral semaglutide 14 mg, the first GLP-1RA to be developed in an oral form, was evaluated in the PIONEER 6 clinical trials. The primary outcome of a major adverse cardiovascular event was noninferior with oral semaglutide to that of placebo. But there were more gastrointestinal adverse events of oral semaglutide, which may lead to poor compliance of patients [75]. Nevertheless, it is worth discussing which kind of preparation will give more clinical benefit in the treatment of type 2 diabetes, oral semaglutide or subcutaneous semaglutide? We tend to attribute the observed cardiovascular benefit of GLP-1RA to glucose-lowering effect. Other aspects include improving insulin resistance, lowering of LDL-c and triglycerides, and reductions body weight.

## 2. Conclusions

As a novel hypoglycemic drug, the GLP-1RA can reduce blood sugar but not cause hypoglycemic events. It further improves myocardial ischemia, inhibits plaque progression, regulates lipid metabolism, reduces myocardial oxygen consumption, improves vascular endothelial function, and reduces weight by reducing oxidative stress, inflammation, apoptosis, and myocardial fibrosis, thereby exerting direct or indirect protective effects on the cardiovascular system. Whether the cardiovascular beneficial effects of GLP-1RA drugs are independent of the hypoglycemic mechanism is still unclear. Further research is required to provide more theoretical basis for the prevention and treatment of cardiovascular diseases.

## Data Availability

There is no raw data associated with this review.
